# Construction of a *mariner*-based transposon vector for use in insertion sequence mutagenesis in selected members of the *Rhizobiaceae*

**DOI:** 10.1186/s12866-014-0298-z

**Published:** 2014-11-30

**Authors:** Benjamin J Perry, Christopher K Yost

**Affiliations:** Department of Biology, University of Regina, 3737 Wascana Parkway, Regina, SK S4S 0A2 Canada

## Abstract

**Background:**

The *Rhizobiaceae* family of Gram-negative bacteria often engage in symbiosis with plants of economic importance. Historically, genetic studies to identify the function of individual genes, and characterize the biology of these bacteria have relied on the use of classical transposon mutagenesis. To increase the rate of scientific discovery in the *Rhizobiaceae* there is a need to adapt high-throughput genetic screens like insertion sequencing for use in this family of bacteria. Here we describe a *Rhizobiaceae* compatible MmeI-adapted *mariner* transposon that can be used with insertion sequencing for high-throughput genetic screening.

**Results:**

The newly constructed *mariner* transposon pSAM_Rl mutagenized *R. leguminosarum*, *S. meliloti*, and *A. tumefaciens* at a high frequency. In *R. leguminosarum*, mutant pools were generated that saturated 88% of potential *mariner* insertions sites in the genome. Analysis of the *R. leguminosarum* transposon insertion sequencing data with a previously described hidden Markov model-based method resulted in assignment of the contribution of all annotated genes in the *R. leguminosarum* 3841 genome for growth on a complex medium. Good concordance was observed between genes observed to be required for growth on the complex medium, and previous studies.

**Conclusions:**

The newly described *Rhizobiaceaee* compatible *mariner* transposon insertion sequencing vector pSAM_Rl has been shown to mutagenize at a high frequency and to be an effective tool for use in high-throughput genetic screening. The construction and validation of this transposon insertion sequencing tool for use in the *Rhizobiziaceae* will provide an opportunity for researchers in the *Rhizobiaceae* community to use high-throughput genetic screening, allowing for significant increase in the rate of genetic discovery, particularly given the recent release of genome sequences from many *Rhizobiaceae* strains.

**Electronic supplementary material:**

The online version of this article (doi:10.1186/s12866-014-0298-z) contains supplementary material, which is available to authorized users.

## Background

Insertion sequencing (INSeq) is a technique for high throughput forward genetic screening that has recently become a favorable approach to studying gene function at the genome scale [1,2]. INSeq relies on the use of next-generation DNA sequencing to audit the presence of hundreds of thousands of unique transposon insertions present in a pool of mutants that collectively saturate that organism’s genome with transposition events [3–6]. In general, INSeq based methods can use two different methods to analyze gene function. The first relies on sequencing the transposon insertions sites in an input pool and an output pool of transposon mutants, and using the differential representation of mutants in each pool to infer the functional role of each gene with sufficient representation of insertion sites [7]. The second method relies on creating a mutant pool sufficiently large and complex that it saturates the genome and allows for analysis of regions with statistically fewer, or no, insertions than expected using a non-parametric [8], Bayesian model [9], or hidden Markov model (HMM) based analysis [10,11]. Both approaches have been applied to several species of bacteria to investigate genes involved in colonization of hosts [12–14], resistance to antibiotics [15], characterizing metabolic pathways [16,17], deducing core essential genomes [18–24], and recently, examining genes involved in colonizing soil environments [7].

The *Rhizobiaceae* is a family of alpha-proteobacteria containing three agriculturally important genera of soil bacteria: *Rhizobium*, *Sinorhizobium* and *Agrobacterium* [25]. Members in these genera share a unique relationship with plant hosts. *Rhizobium*, and *Sinorhizobium* are both able to enter into an endosymbiotic mutualism with certain species of leguminous plants, in which the Rhizobia fix atmospheric nitrogen into a biologically available form for the plant in return for fixed carbon and energy [26]. This symbiosis is particularly important in the context of agriculturally produced pulse crops, where the Rhizobium legume symbiosis affords farmers the ability to reduce the rate of synthetic nitrogen fertilizers application [27]. Conversely, the relationship of *Agrobacterium* with its plant host is parasitic. In this symbiosis, *Agrobacterium* infects the tissues of a plant host and transforms specific virulence genes into the host’s DNA, resulting in tumorgenic growth with altered cellular metabolism that the bacteria then colonize [28]. The formation of several galls at the stem root interface results in a plant infection known as crown gall, that can have a significant impact on the crop yield of stone fruits, berries, and nuts [29].

Genetic research in *Rhizobium*, *Sinorhizobium*, and *Agrobacterium* has relied heavily on the use of transposon mutagenesis screens. Perhaps the most commonly used transposon in the *Rhizobiaceae* is the Tn5 transposon [30–32]. The use of Tn5 genetic screens is numerous and has helped to elucidate genes involved in metabolism [33–35], desiccation tolerance [36,37], and cell envelope physiology [38] for example. Implementation of transposon mutagenesis with the high-throughput techniques of INSeq promise to accelerate the rate at which genetic research in the *Rhizobiaceae* is currently performed. Furthermore, it would allow for comprehensive genome screens for genes involved in host interactions, metabolism, survival, and possibly plasmid maintenance, under any testable condition.

The *mariner* class of transposon is a host independent transposon that unlike the random insertion transposons such as Tn5 is known to specifically insert into an organism's genome at thymine-adenine (‘TA’) motifs [39]. Because of this defined insertion preference, transposition events can be modeled *in silico* in any sequenced genome to understand the defined number of insertion locations that exist. This type of analysis can be further refined to examine insertions per gene or within any defined region of interest in the genome. Furthermore, using a transposon with a defined number of insertion loci allows for robust statistical analysis when used in a transposon insertion sequencing methodology. One such analysis package uses a HMM to predict the essentiality of every gene under a particular growth condition in an organism’s genome given a sufficiently dense INSeq data set [10]. The advantages of this type of analysis is that it does not solely rely on the comparison of an input and output pool of mutants as it uses statistical inference on a defined number of insertion sites to infer an over represented or under represented number of insertions within a given region.

Here we describe the modification of a previous INSeq mutagenesis vector pSAM_Bt, which uses a mariner class transposon with modified IR elements, to selectively capture 15–16 bp of genomic DNA adjacent to the transposon insertion [4]. The DNA fragment is then processed for next-generation DNA sequencing. The new INSeq vector uses a *Rhizobiaceae* specific promoter to drive the expression of the plasmid-borne transposase, and has had the erythromycin resistance gene cassette replaced with a neomycin resistance gene cassette (*ntpII)* for ease of selection in the *Rhizobiaceae*. The host range of transposition with pSAM_Rl was examined and an INSeq experiment was performed on the model organism *Rhizobium leguminosarum* bv. *viciae* 3841 (RLV3841) to examine the insertion density that the pSAM_Rl transposon could achieve. Furthermore, the functionality of the transposon in RLV3841 for INSeq was also examined.

## Results

### Construction of pSAM_Rl and transposition frequency within the *Rhizobiaceae*

**Figure 1:**
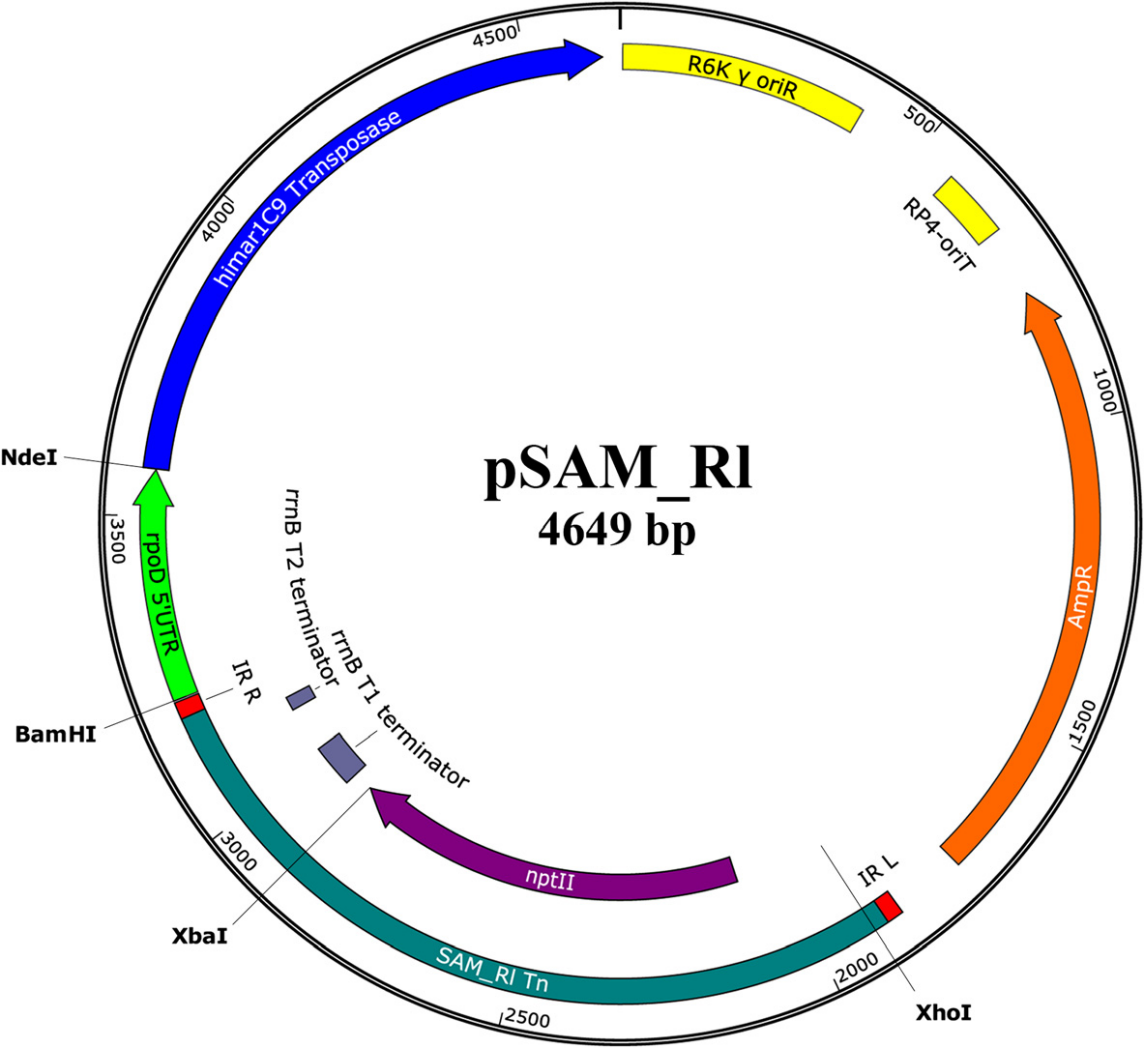
**pSAM_Rl plasmid map.** Restriction enzymes used for cloning are indicated. Antibiotic markers (ampicillin, AmpR; neomycin/kanamycin, *nptII*), origin of replication (R6K y oriR), origin of transfer (RP4-oriT), transposase (himar1C9), tranposase promoter (*rpoD* 5'UTR), MmeI-adapted *mariner* inverse repeats (IR_R, IR_L), transposon borne Rho-independant terminator (*rrnB* T1, *rrn*B T2). Plasmid map produced using Snapgene software.

The MmeI-adapted *mariner* transposon suicide delivery vector pSAM_Rl was constructed from a previously described MmeI-adapted *mariner* delivery vector pSAM_Bt [4]. To do so, the 974 bp Ery^R^ in pSAM_Bt was replaced with a 979 bp Neo/Kan^R^ resistance cassette from pSC189 and the 304 bp *Bacteroides thetaiotaomicron rpoD* promoter region was replaced with a 366 bp RLV3841 *rpoD* promoter region. A map of the pSAM_Rl construct is shown in Figure 1, with restriction enzyme sites used for cloning indicated. The pSAM_Rl construct was maintained in *E. coli* strain SM10λ*pir* as this strain harbours both the λ*pir* gene, required for the replication of R6Kƴ *oriR*, and chromosomally integrated *tra* genes, required for conjugative transfer via the plasmid borne RP4-*oriT*. Analysis of the transposition frequency of the pSAM_Rl *mariner* transposon was evaluated via conjugative transfer of the pSAM_Rl suicide vector from *E. coli* SM10λ*pir* into *R. leguminosarum, S. meliloti, and A. tumefaciens*. Transposition frequency was highest in RLV3841, yielding an average of 2.01 × 10^−4^ transposon mutants per recipient cell. The frequencies of transposition in *A. tumefaciens* and *S. meliloti* were observed to be 8.04 × 10^−5^ and 2.54 × 10^−5^ mutants per recipient cells, respectively.

### Analysis of pSAM_Rl transposition in the RLV3841 genome

Analysis of pSAM_Rl transposon integration was performed using three independent pools of ~1.9 × 10^6^ transposon mutants. Mutant pools were grown for approximately 16 generations (48 hours) on TY basal medium. The mutants were collected *en masse* and the 15–16 bp of genomic DNA adjacent to the transposon insertion was sequenced using a modified INSeq method on the Ion Torrent PGM. Transposon insertion sequences from the three replicate samples were then pooled and aligned onto the RLV3841 genome, resulting in 2,319,239 usable transposon insertion tags (Tn-tags) for analysis with the transposon HMM (Tn-HMM) python module [10]. From *in silico* analysis it was calculated that 140,056 potential *mariner* transposon insertion sites existed within the RLV3841 genome. Of these, 116,544 unique transposon insertions were observed in the composite data collected from the three pSAM_Rl mutant pools grown on TY agar. This corresponded to an overall insertion density of 83% in the RLV3841 genome, with insertion density across replicons ranging between 68% to 88% (Table 1). The mean read count per *mariner* insertion site, observed to have a transposon insert, ranged from 18.57 to 22.09 sequence reads per insertion across all replicons with the median read count per insertion ranging from 10.0 to 13.0 reads per insertion (Table 1).

**Table 1:** **Summary of pSAM_Rl transposition in RLV3841**

**Replicon**	**Size (bp)**	**GC%**	**Gene number**	**Potential insertions**	**Observed insertions**	**Insertion density** ^**A**^	**Mean read count** ^**B**^	**Median read count** ^**C**^
Chrom	5057142	61.10	4800	89660	73375	0.82	20.46	13.00
RL12	870021	61.00	790	14845	13122	0.88	18.57	12.00
RL11	684202	61.00	644	12456	10964	0.88	19.97	13.00
RL10	488135	59.60	471	10003	8470	0.85	17.19	10.00
RL9	352782	61.00	313	6359	5453	0.86	19.65	13.00
RL8	147463	58.70	142	3118	2702	0.87	17.96	11.00
RL7	151546	57.60	186	3615	2458	0.68	22.09	12.00
Genome	7751291	60.00	7346	140056	116544	0.83	19.41	12.00

^A^Insertion Density is the fraction of the 'TA' insertions sites that contain a Tn insertion.

^B^Mean Read Count is the mean number of Tn insertions within 'TA' sites.

^**C**^Median Read Count is the median number of Tn insertions within a specific 'TA' site.

### Distribution of phenotypic classes throughout the RLV3841 genome

Each RLV3841 replicon was analyzed using the Tn-HMM python module [10] to classify genes based on the observed density of Tn insertions in each gene within the mixed mutant cell population. For example, a gene which had no detectable Tn insertion sites following DNA sequencing from the pooled mutant DNA suggests cells carrying a mutation in this gene were not maintained in the population. These genes are described as conferring an essential phenotype. The procedure is described in greater detail in the Materials and Methods.

The seven replicons of RLV3841 were analyzed separately to account for variations in mean read depth, insertion density, and median read count between the replicons that may have been a result of their independent replication within the cell. Across all replicons 89.5% of the genes were observed to have a neutral or negligible impact on the ability of RLV3841 to grow on TY medium when disrupted by the transposon (Table 2). Genes identified as conferring a phenotype classification of essential, growth-defective, and growth-advantage were observed to represent 5.6%, 4.0%, and 0.1% of the genes within the genome, respectively. Throughout the genome, 10 genes contained no 'TA' transposon insertions sites and as such could not be assigned to a growth effect state. However this represents only 0.14% of the total predicted genes within the genome (Table 2).

**Table 2:** **Summary of phenotypic classes across the RLV3841 genome**

**Replicon**	**Mapped reads**	**Genes per specific phenotypic class** ^**A**^				
		**ES**	**GD**	**NE**	**GA**	**No Data** ^**B**^
Chrom	1501032	317	272	4176	35	0
pRL12	243699	8	3	774	3	2
pRL11	218944	15	9	614	3	3
pRL10	145621	15	3	447	3	3
pRL09	107144	7	1	305	0	0
pRL08	48512	3	1	137	1	0
pRL07	54287	44	8	124	8	2

ES = Essential, GD = Growth-defective, NE = Neutral, GA = Growth-advantage.

^A^Genes were designated to a phenotypic class based on the state most frequently observed in the TA sites within the boundaries of an annotated gene, except in the case of essential genes which could also have been assigned to the ES state if they contained a stretch of ES TA sites that was statistically significant based on the extreme value distribution. Please refer to the Materials and Methods section for more detail.

^B^Genes lacking TA motifs.

### Specific essential genes within the RLV3841 genome

The defined phenotypic class of several house-keeping genes involved in ATP synthesis, cell divisions, DNA replication, and RNA transcription are summarized in Table 3. Additionally, Table 3 summarizes the essential nature of plasmid specific replication genes for each of the 6 plasmids contained in the RLV3841 genome that are required for plasmid maintenance during cell division. From previous analysis [38], genes known to be required for growth on TY medium are summarized at the end of Table 3 with their essential nature, as determined by the 4 phenotypic classes, reported by the Tn-HMM software.

**Table 3:** **Selected genes in RLV3841 cultured on TY agar with Tn insertions substantially below expected levels**

**Locus**	**Gene**	**Gene annotation**	**Potential insertions** ^**A**^	**Insertion density** ^**B**^	**Mean read count** ^**C**^	**Phenotypic class** ^**D**^
**Selected chromosomal house keeping genes**						
RL0924	*atpI*	Putative ATP synthase I	4	0.000	0.000	ES
RL0925	*atpB*	F0F1 ATP synthase subunit A	12	0.083	1.000	ES
RL0926	*atpC*	F0F1 ATP synthase subunit C	7	0.000	0.000	ES
RL0927	*atpG*	F0F1 ATP synthase subunit B'	7	0.000	0.000	ES
RL0928	*atpF*	F0F1 ATP synthase subunit B	4	0.000	0.000	ES
RL4405	*atpC*	F0F1 ATP synthase subunit Epsilon	3	0.333	1.000	ES
RL4407	*atpD*	F0F1 ATP synthase subunit Beta	23	0.043	1.000	ES
RL4408	*atpG*	F0F1 ATP synthase subunit Gamma	12	0.083	1.000	ES
RL4409	*atpA*	F0F1 ATP synthase subunit Alpha	22	0.000	0.000	ES
RL4410	*atpH*	F0F1 ATP synthase subunit Delta	8	0.125	1.000	ES
RL4412	*priA*	Primosome assembly protein PriA	23	0.261	4.000	GD
RL3408	*dnaG*	DNA primase	23	0.000	0.000	ES
RL3298	*ftsZ*	Cell division protein FtsZ	9	0.000	0.000	ES
RL3299	*ftsA*	Putative cell division protein FtsA	12	0.083	1.000	ES
RL3300	*ftsQ*	Putative cell division protein FtsQ	14	0.000	0.000	ES
RL3308	*ftsW*	Putative cell division protein FtsW	13	0.000	1.000	ES
RL3965	*ftsH*	Putative cell division protein FtsH	19	0.105	1.500	ES
RL2515	*gyrB*	DNA topoisomerase IV subunit B	26	0.115	9.333	ES
RL2401	*gyrA*	DNA gyrase subunit A	34	0.059	1.000	ES
RL1723	*dnaE*	DNA polymerase III subunit Alpha	51	0.078	1.000	ES
RL4697	*dnaE*	Putative DNA polymerase III subunit Alpha	76	0.961	23.562	NE
RL0334	*dnaN*	DNA polymerase III subunit Beta	14	0.071	1.000	ES
RL2099	*recJ*	Putative single-stranded-DNA-specific exonuclease	17	0.294	3.800	GD
RL1766	*rpoB*	DNA-directed RNA polymerase subunit Beta	64	0.016	1.000	ES
RL1767	*rpoC*	Putative DNA-directed RNA polymerase subunit Beta'	61	0.066	1.750	ES
RL1798	*rpoA*	Putative DNA-directed RNA polymerase subunit Alpha	12	0.000	0.000	ES
RL0059	*-*	Putative ATP-dependant helicase	18	0.778	21.714	NE
RL0582	*-*	Putative ATP-dependant helicase	39	0.949	18.730	NE
RL1551	*dnaC*	Putative replicative DNA helicase	24	0.042	24.000	ES
**Selected plasmid borne genes**						
pRL120001	*repA*	Putative replication protein A	21	0.000	0.000	ES
pRL120002	*repB*	Putative replication protein B	9	0.000	0.000	ES
pRL120003	*repC*	Putative replication protein C	13	0.000	0.000	ES
pRL110001	*repA*	Putative replication protein A	25	0.040	1.000	ES
pRL110002	*repB*	Putative replication protein B	16	0.063	1.000	ES
pRL110003	*repC*	Putative replication protein C	15	0.067	1.000	ES
pRL100001	*repA*	Putative RepA replication protein	19	0.000	0.000	ES
pRL100002	*repB*	Putative RepB replication protein	6	0.000	0.000	ES
pRL100003	*repC*	Putative RepC replication protein	22	0.045	1.000	ES
pRL90001	*repA*	Putative replication partitioning protein	30	0.000	0.000	ES
pRL90002	*repB*	Putative replication partitioning protein	19	0.053	1.000	ES
pRL90003	*repC*	Putative replication initiation protein RepC	15	0.000	0.000	ES
pRL80001	*repA*	Putative replication protein RepA	38	0.000	0.000	ES
pRL80002	*repB*	Putative replication protein RepB	23	0.043	1.000	ES
pRL80003	*repC*	Putative replication initiation protein RepC	28	0.036	1.000	ES
pRL70092	*repA*	Putative replication protein	34	0.529	1.722	GD
pRL70093	*repB*	Putative replication protein B	22	0.455	1.100	GD
pRL70094	*repC*	Putative replication initiation protein RepC	33	0.455	1.200	GD
**Previously experimentally confirmed genes with a TY- defective phenotype**						
RL4692	*ctpA*	Putative carboxy-terminal processing protease precursor	14	0.429	5.500	GD
RL3501	-	Conserved hypothetical membrane protein	42	0.667	3.107	GD
RL2815	*fabF2*	3-Oxoacyl acyl carrier protein synthase	11	0.364	1.000	GD
RL1375	*phaD2*	Putative Na+/H+ antiporter subunit D	26	0.192	1.400	GD
**Examples of genes with higher than expected Tn insertions** ^**E**^						
RL0868	-	Putative lipid A oxidase	16	1.000	113.688	GA
RL2661	-	Putative transmembrane component of ABC transporter	13	1.000	62.846	GA
RL0684	-	Putative transmembrane protein	27	0.963	64.346	GA

ES = Essential, GD = Growth Defect, NE = Neutral, GA = Growth Advantage.

^A^Potential insertions is the number of 'TA' nucleotide motifs within the gene.

^B^Insertion density is the fraction of all 'TA' insertions sites with a Tn insertion.

^C^Mean read count is the mean number of Tn insertions at 'TA' sites with a Tn insertion.

^D^Genes were designated to a phenotypic class based on the state most frequently observed in the TA sites within the boundaries of an annotated gene, except in the case of essential genes which could also have been assigned to the ES state if they contained a stretch of ES TA sites that was statistically significant based on the extreme value distribution. Please refer to the Materials and Methods section for more detail.

^E^Three genes with the phenotypic classification of Growth-advantage were included to provide context to the insertional densities and mean read count of the Growth-advantage state compared to the essential state classification.

## Discussion

High throughput forward genetic screening is rapidly being adopted in a diverse range of organisms. INSeq, and similar high-throughput techniques, have been used in several bacterial species, including *Salmonella* [5], *Mycobacterium* [17], *Haemophilus* [6], *Vibrio* [40], *Pseudomonas* [15]*, Chronobacteria* [24] and *Bacillus* [7]. The technique is very attractive as it allows for the high-throughput functional screening of an organism's genome under varied growth conditions. Here we present the design and validation of a transposon mutagenesis system that will allow for the application of high-throughput INSeq genetic screening for use within the *Rhizobiaceae*.

### Transposon mutagenesis with pSAM_Rl in RLV3841

We have successfully demonstrated the implementation of a *mariner* class transposon to mutagenize selected species within the family *Rhizobiaceae.* We found that the MmeI-adapted *mariner* transposon harbored on pSAM_Rl could mutagenize *R. leguminosarum*, *A. tumefaciens*, and *S. meliloti* at a high frequency. In the case of RLV3841, it was observed that this high frequency of mutagenesis was dense enough to generate saturating libraries of transposon insertion mutants. We were able to generate transposon mutant libraries that saturated the RLV3841 genome with an insertion density of 0.88, which is higher then the insertion density of the data set used for validation of the HMM used for analysis [10]. This suggests that the combination of *mariner* based transposon insertion sequencing with the Bayesian based HMM analysis would yield accurate and full genome level results. Furthermore, our analysis used approximately 2.3 M reads. And increased read depth could help increase confidence in the analysis, particularly in resolving the phenotypic classifications of essential and growth defective.

We also observed no bias of *mariner* transposon insertion across the RLV3841 chromosome and plasmids, suggesting that the transposon inserts randomly and therefore should allow for reliable whole-genome screening approaches. Analysis of read depth showed that there was also little bias in mean read-count per insertion, except in the case of pRL7, which was slightly higher than the other replicons. We suggest that this is a result of pRL7 being maintained at a higher copy number than the other megaplasmids and chromosome, and so sampling of the mutant pool DNA would results in a higher sampling of pRL7 transposon insertion tags than the other plasmids, or chromosome. When the increase in mean read counts is taken into consideration with the insertion density, it appears that although the higher copy number of pRL7 resulted in a higher mean read depth, it did not increase the insertions density, suggesting that the saturation of pRL7 had reached a plateau and an increased presence of pRL7 did not result in a corresponding increase in the number of unique insertions sites.

### Analysis of RLV3841 INSeq using a hidden Markov model

Analysis of the TY INSeq data set with the Tn-HMM analysis package assigned accurate phenotypic classification to several genes thought to be essential housekeeping genes necessary for growth under normal conditions. The analysis showed that insertions in genes required for ATP synthesis were absent in the mutant pools. These genes are expected to be essential due to their central role in metabolism and their designation as essential in this assay supports the validity of using the MmeI-adapted *mariner* in conjunction with the HMM analysis as our INSeq methodology. Furthermore, visual investigation of the transposon insertion density around the region encoding the ATP synthase genes revealed a high insertion density leading up to and after the genes, further supporting the conclusion that the technique is robust and can discern regions of essentiality from those of other states at a high resolution.

In a few instances, a gene expected to be essential was observed to be neutral. RL4697 is annotated as a putative DNA polymerase III alpha sub-unit, and is therefore predicted to be required for proper DNA polymerase function; however, the gene was classified in the neutral category with 96.1% of all potential insertion sites in the locus observed to have insertions. We suggest that RL4697 may be misanotated, as RL1723, another DNA polymerase III alpha subunit, was observed to be essential (Table 3). This highlights another potential use of INSeq in the *Rhizobiaceae* for validation and quality improvement of genome annotations.

Five of the megaplasmids in the RLV3841 genome were observed to have a set of 3 plasmid replication genes that the INSeq approach identified as essential for plasmid replication and maintenance. The exception was pRL7 which has two sets of replication genes [41] and therefore functional redundancy may have complicated the the classification of the pRL7 *rep* genes into the phenotypic classes. The classification of the *rep* genes on each plasmid as essential provides validation of the INSeq method. Tn insertion within a replication locus would result in the loss of the plasmid from the mutant cell populations harvested for DNA extraction and INSeq DNA sequencing. This result highlights the value of the INSeq approach in the genetic characterization of novel plasmids, as the method is able to identify plasmid encoded genes that are required for plasmid replication and maintenance. Furthermore the method can identify plasmid-encoded genes that provide a fitness advantage to the host under specific growth conditions based on an observed low Tn insertion density.

The genes from previously described mutants with growth defects on TY medium were also examined. Previous work identified [38] several genes that are important for growth on TY medium. When we compared those results with the results produced by the INSeq analysis of RLV3841 grown on TY medium we observed good concordance with these previously published results. In our results, the four TY related genes were all observed to result in a growth-defective phenotype after ~16 generations of growth, which is in agreement that the interruption of these genes via mutagenesis will result in impaired growth on TY.

## Conclusions

The construction and validation of the *mariner* pSAM_Rl transposon delivery vector as a transposon insertion sequencing tool for use in the *Rhizobiziaceae* will provide an opportunity for researchers in the *Rhizobiaceae* community to use a new high throughput genetic screening approach. There are many research opportunities within the *Rhizobiaceae* that could be examined using the INSeq methodology. For example, use of a INSeq approach in rhizobia to study genes required for rhizosphere colonization and plant infection will help to understand the competition problem observed in inoculant strains, by not only identifying new essential rhizosphere colonization genes, but also identifying mutations that provide a phenotypic growth advantage . Furthermore, the use of INSeq could be used to fully elucidate catabolic pathways, if saturating mutant pools were grown on minimal medium given a single carbon source, and contrasted with the results of similar experiments on rich media. In the near term, we will use the INSeq approach to increase our understanding of the gene networks involved in swarming physiology in RLV3841.

## Materials and Methods

### Bacterial strains, growth conditions and plasmids

The bacterial strains and plasmids used in this study are presented in Table 4. *R. leguminosarum*, *S. meliloti*, and *A. tumefaciens* were cultured at 30°C using tryptone-yeast extract medium (TY) [42]. *E. coli* strains were cultured on lysogeny broth (LB) at 37°C [43]. When required, antibiotics were used at the following concentrations for *Rhizobiaceae* 500 μg/mL streptomycin (Str), 50 μg/mL rifampicin(Rif), 50 μg/mL neomycin (Neo); concentrations used for *Escherichia coli* were 100 μg/mL ampicillin (Amp), 25 μg/mL erythromycin, and 50 μg/mL kanamycin. The plasmid pSAM_Bt was obtained as a gift from Dr. Andrew L. Goodman. Plasmid pSC189 was obtained from addgene.org (plasmid#: 32114) as kindly directed by Dr. Eric J. Rubin.

**Table 4:** **Summary of bacterial strains and plasmids**

**Strains**	**Characteristics**	**Reference**
*R. leguminosarum bv. viciae 3841*	Str^R^ wildtype	[44]
*S. meliloti RM1021*	SU47 str-21 Str^R^	[45]
*A. tumefaciensUBAPF2*	Plasmid-free derivative of *A. tumefaciens* strain C5 Rif^R^	[46]
*E. coli SM10 λpir*	*thi-1 thr leu tonA lacY supE recA::RP4-2-Tc::Mu KanR λpir*	[47]
*E. coli PIR1*	F- ∆*lac169* *rpoS*(Am) *robA1* *creC510 hsdR514 endA- recA1 uidA*(∆MluI)::pir-116	Invitrogen
**Plasmids**		
pSAM_Bt	Amp^R^ Ery^R^; RP4-oriT, oriR6K, mariner himar1C9 transposase with *Bacteroides thetaiotamicron* rpoD promoter, MmeI-adapted marinerIR elements	[4]
pSC189	Amp^R^ Kan^R^; RP4-oriT, oriR6K	[48]
pGEM-T Easy	Amp^R^ ; cloning vector	Invitrogen
pGEM::189KmR	Amp^R^ Kan^R^; pGEM-T vector containing PCR amplified nptII gene from pSC189	This Study
pGEM::rpoD	Amp^R^; pGEM-T vector containing PCR amplified *R. leguminosarum* 3841 rpoD promoter region	This Study
pSAM_Km	Amp^R^ Kan^R^; Ery^R^ in pSAM_Bt replaced with nptII from pSC189	This Study
pSAM_Rl	Amp^R^ Kan^R^; pSAM_Km with *B. thetatiotamicron* rpoD promoter replaced with *R. leguminosarum* 3841 rpoD promoter region	This Study

### Construction of pSAM_Rl

Plasmid DNA was isolated using GenElute™ Plasmid Miniprep Kit (Sigma-Aldrich). The Neo/Kan^R^ cassette within pSC189 [48] was PCR amplified using primers Tn189KmR_Fwd_XhoI and Tn189_Rev_XbaI (Additional file 1: Table S1) such that the Xho*I* and Xba*I* restriction enzyme sites were introduced on the 5’ and 3’ end, respectively. The 991 bp Neo/Kan^R^ PCR product was subsequently cloned into the pGEM®-T Easy Vector System creating plasmid pGEM::189Km^R^. The Neo/Kan^R^ cassette in pGEM::189Km^R^ was digested with Xho*I* and Xba*I* restriction enzymes creating a 979 bp fragment that was directionally cloned into the pSAM_Bt *mariner* transposon. The resulting plasmid pSAM_Km was maintained in the *E. coli* strain PIR1(Invitrogen), which allows for high copy number maintenance of R6Kƴ-oriR plasmids.

Cloning of the RLV3841 *rpoD* promoter region was carried out by PCR amplifying a 366 bp region upstream of the *rpoD* gene start codon using primers Rlv_rpoD_Pro_Fwd and Rlv_rpoD_Pro_Rev (Additional file 1: Table S1). The *rpoD* promoter PCR product had a 5’ *BamHI* and 3’ Nde*I* restriction enzyme site introduced, and was subsequently cloned into the pGEM®-T Easy Vector System to create the plasmid pGEM::rpoD. The RLV3841 *rpoD* promoter region was then excised from pGEM::rpoD using Nde*I* and Bam*HI*, and directionally cloned into pSAM_Km to create the vector pSAM_Rl (Figure 1). The new pSAM_Rl suicide vector carried an MmeI-adapted *mariner* transposon harbouring a Neo^R^/Kan^R^ cassette, and had the himar1C9 transposase transcriptionally fused to a RLV3841 *rpoD* promoter. For use in transposon mutagenesis, the pSAM_Rl construct was electroporated into *E. coli* SM10λ*pir* (obtained from Dr. Peter Howard, University of Saskatchewan).

### Testing pSAM_Rl transposition frequency

Transposition mutagenesis using pSAM_Rl was done in triplicate. Donor and recipient cells were grown in broth culture to an OD_600_ of approximately 0.8 and were pooled in a ratio of 1000 μL recipient to 500 μL of donor in a 1.5 mL microcentrifuge tube. The conjugation mixture was pelleted at 12,500 rpm for 3 min, washed once with 1000 μL 1X PBS, and resuspended in approximately 100 μL 1X PBS. The cell suspensions were then spotted onto pre-warmed TY agar plates and incubated at 30°C overnight. Conjugation spots were scraped and resuspended in 1000 μL of 1X PBS. Enumeration of transposon mutants was done using TY agar supplemented with 50 μg/mL Neo and the appropriate *Rhizobiaceae* counter selectable antibiotic (Table 4). Enumeration of total *Rhizobiaceae* was done on TY agar with the species specific selectable antibiotic.

### Generating transposon mutant libraries for sequencing

Six independent conjugations of pSAM_Rl into RLV3841 were conducted on TY agar as described above. After 24 hours incubation at 30°C each of 6 mating spot was scraped and resuspended in 1 mL of 1X PBS and then pooled together in a final volume of 6 mL. For selection on TY agar, 1000 μL of resuspended cells were plated across 2 separate 245 × 245 mm^2^ (Corning) Neo + Str TY agar plates, in triplicate and incubated for ~48 hour at 30°C.

Following incubation, a faint film like growth was scraped off each plate and resuspended in 1 mL of 1×PBS, vortexed for 1 minutes, and then pelleted at 15, 000 RPM for 10 minutes. The supernatant of each resuspension was very viscous and still contained cells, it was equally aliquoted into 2 × 1.5 mL microcentrifuge tubes. The original pellet, and two tubes of supernatant, were then brought up to a final volume of 1000 μL with 1 M NaCl, vortexed thoroughly, and incubated on ice for 1 h to disrupt the viscous exopolysaccharide diffuse capsule to better collect the cells. The NaCl cell suspensions were then pelleted at 15, 000 RPM for 10 minutes, and the pellets from each replicate were pooled independently and resuspended in 1000 μL of TE buffer (pH 8.0). The resulting 3 mutant pools were used for independent DNA isolation and downstream library preparation.

### Preparing sequencing libraries and DNA sequencing

Transposon insertion tags (Tn-tags) consisted of 53 bp of pSAM_Rl transposon sequence, including the 27 bp inverse repeat sequence, and 15–16 bp of adjacent genomic DNA. Library preparation was carried out independently for each of the 3 collected Tn-mutant pools. Tn-tags were prepared for DNA sequencing using a modified version of the INSeq method [49] to make the sequencing process amendable to the Ion Torrent PGM sequencing platform. Linear PCR products were amplified using the primer *Ion Torrent BioSAM* (Additional file 1: Table S1), with an annealing temperature of 58.6°C and 500 ng of template DNA. Linear PCR products were purified using a QIAquick PCR Purification Kit (Qiagen) according to the manufacturer's recommended protocol. The biotinylated linear PCR products were then bound to Pierce Streptavidin Magnetic Beads (Thermo Scientific) and enzymatic steps during library preparation were performed as described [49] with the substitution of Klenow (New England Biolabs), Random Primer 6 (New England Biolabs) and T4 DNA Ligase (New England Biolabs). Additionally, a custom library adapter, *INSEQ_Adpt*, was used in the adapter ligation step. The final PCR amplification of sequencing template was accomplished using fusion primers designed in accordance with Ion Amplicon Library Preparation (Fusion Method, Pub#: 4468326 Rev. C), using the PCR amplification conditions described in the INSeq methodology [49]. The forward fusion primers *IT_A_FP_1, IT_A_FP_2,* and *IT_A_FP_3* included IonXpress barcode sequences 1, 2, and 3 respectively, for downstream sequence separation. The reverse primer *IT_trP1_FP* was used in conjunction with a forward primer to introduce the trP1 sequencing adapter. The final sequencing template prepared from the Ion Amplicon library preparation was 187 bp in length. Sequencing template was gel purified using the Invitrogen E-Gel® SizeSelect™ system, and was analyzed using a Bioanalyzer High Sensitivity DNA Chip (Agilent Technologies) prior to sequencing for quality and molarity. The three technical replicates had a final concentration of 1.37, 1.82, and 1.23 μg/ul of sequencing library after size selection, respectively.

DNA sequencing was performed on the Ion Torrent PGM using 200 bp sequencing chemistry and a 316v2 sequencing chip. The total raw sequencing output of the Ion Torrent was 1.25, 1.73, and 1.74 million reads for each of the 3 replicates. The raw sequencing reads were then pooled for downstream data extraction an analysis.

### Data extraction and transposon insertion analysis

**Figure 2:**
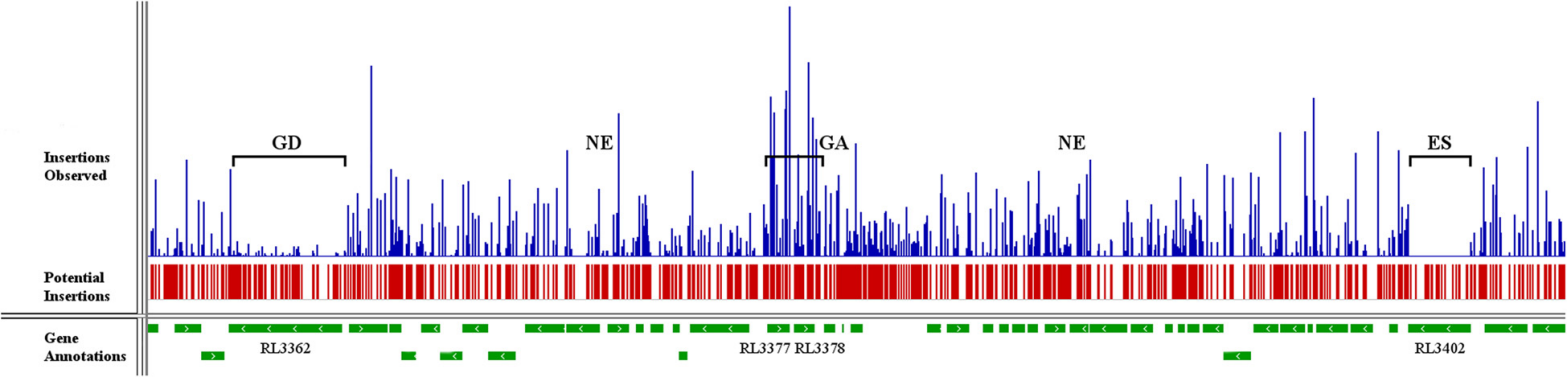
**Transposon insertion density across a selected region of the RLV3841 genome.** growth-defective, neutral, and growth-advantage regions of the RLV3841 genome. Analyzing the total number of insertions mapped to each potential *mariner* insertion site using the Tn-HMM python module [10] allows each gene to be placed in one of four phenotypic classes: ES = essential, GD = growth defect, NE = neutral, GA = growth advantage. In this experiment the phenotype refers to the ability to grow on TY agar. Data visualization was obtained using Integrative Genomics Viewer software [54]. Please refer to the Materials and Methods section for more detail on the process of assigning phenotypic classifications.

Quality trimming to Q20 and trimming of adapter sequences was performed using cutadapt [50] and the final 16–15 bp tn-tags were checked for a leading 'TA' motif using a custom python script. The resulting 3,192,486 transposon insertion tags were mapped to the *R. leguminosarum* bv. *viciae* 3841 reference genome (RefSeq:NC_008378.1 to NC_008384.1) [51] using the Bowtie short read aligner [52], allowing for no mismatches in the alignment, and only reporting insertion tags that mapped to a single unique location. Short read alignment resulted in 2,319,239 unique transposon insertion tags mapping to the RLV3841 reference genome, after 131,676 reads were ommitted due to multiple alignments, and 509,341 reads failed to align. The .sam output file from the Bowtie alignment was converted into a .bam format using Samtools [53], and was then converted to .bed format using bedtools. Transposon insertion reads were grouped by specific RLV3841 replicons for downstream analysis. The .bed files of the aligned transposon insertion tags were converted to .wig format using a custom python module developed in house. The .wig formatted INSeq data sets generated for each of the 7 replicons in the RLV3841 genome were then analyzed independently using the Tn-HMM python module [10]. Briefly, the python module used a HMM as described in [10], in conjunction with the Viterbi algorithm to calculate the state of each 'TA' insertion site within the genome, independent of gene boundaries. Next, the computer module analyzed the state of successive 'TA' sites within gene boundaries to assign a state for the gene as a whole (See Additional file 2 for the RLV3841 chromosomal output). Four phenotypic classifications are possible: essential, growth defective, neutral, and growth advantage. Figure 2 provides a visual example of the four phenotypic classifications found within a selected region of the RLV3841 genome.

## Additional files

Additional file 1: Table S1. Primers and adapter sequences. Additional file 2:
**Phenotypic classes (ES, GD, NE, GD) of RLV3841 chromosomal genes assessed during growth on TY medium, predicted using the Tn-HMM python module (Additional file 2.csv).** The output of the Tn-HMM python module which provides the INSeq data using pSAM_Rl to screen RLV3841 for growth on tryptone-yeast extract media.
