# Supplementary material for: Construction of a mariner-based transposon vector for use in insertion sequence mutagenesis in selected members of the Rhizobiaceae
Source: BMC Microbiol. 2014 Nov 30;14:298. doi: 10.1186/s12866-014-0298-z (PMC4255674; doi:10.1186/s12866-014-0298-z)
Supplement: Additional file 1: Table S1. — Primers and adapter sequences. [file 12866_2014_298_MOESM1_ESM.docx]

| **Additional file 2: table 1. primer and adapter sequences** | | | | | | |
| --- | --- | --- | --- | --- | --- | --- |
| **Name** |  | **Sequence 5' → 3'** |  | **Annealing Temp.** |  | **Reference** |
| Tn189KmR_Fwd_XhoI |  | GCCCTCGAGTAGACTGGGCGGTTTTATGG |  | 57°C |  | This Study |
| Tn189KmR_Rev_XbaI |  | GCGTCTAGAAGTCCCGCTCAGAAGAACTC |  | 57°C |  | This Study |
|  |  |  |  |  |  |  |
| Rlv_rpoD_Pro_Fwd |  | CATATGGGTTGCCATTTATATCGTCGC |  | 58°C |  | This Study |
| Rlv_rpoD_Pro_Rev |  | GGATCCATAAGGGTTCGCAATCGCTGC |  | 58°C |  | This Study |
|  |  |  |  |  |  |  |
| pSAM_Rl_Conf_Fwd |  | GCTAGACTGGGCGGTTTTATG |  | 62°C |  | This Study |
| pSAM_Rl_Conf_Rev |  | CTGCAGGTAGAAACGCAAAAAG |  | 62°C |  | This Study |
|  |  |  |  |  |  |  |
| Ion Torrent BioSAM |  | /BiotinTEG/CGGTTCGCTTGCTGTCCATAAAAC |  | 58.6°C |  | This Study |
|  |  |  |  |  |  |  |
| M12 Top |  | CTGTCCGTTCCGACTACCCTCCCGAC |  | - |  | Goodman et al. (2009) |
| M12 Bottom |  | GTCGGGAGGGTAGTCGGAACGGACAG |  |  |  | Goodman et al. (2009) |
|  |  |  |  |  |  |  |
| INSeq_Adpt_Top |  | AGATCGGAAGAGCGTCGTGTAGGCAA |  | - |  | This Study |
| INSeq_Adpt_Bottom |  | TTCCCTACACGACGCTCTTCCGATCTNN |  |  |  | This Study |
|  |  |  |  |  |  |  |
| IT_A_FP_1 |  | CCATCTCATCCCTGCGTGTCTCCGACTCAG CTAAGGTAAC GATATAAAACCGCCCAGTCTACTCGAGGG |  | 62°C |  | This Study |
| IT_A_FP_2 |  | CCATCTCATCCCTGCGTGTCTCCGACTCAG TAAGGAGAAC GATATAAAACCGCCCAGTCTACTCGAGGG |  | 62°C |  | This Study |
| IT_A_FP_3 |  | CCATCTCATCCCTGCGTGTCTCCGACTCAG AAGAGGATTC GATATAAAACCGCCCAGTCTACTCGAGGG |  |  |  |  |
| IT_trP1_FP |  | CCTCTCTATGGGCAGTCGGTGATTTCCCTACACGACGCTCTTCCGATCT |  | 62°C |  | This Study |
|  |  |  |  |  |  |  |

**Supplementary Figure 1. Transposon insertion density across essential, growth-defective, neutral, and growth-advantage regions of the RLV3841 genome.** The total number of reads mapped to each potential *mariner* insertion site, and the corresponding state of each region indicated amongst a neutral background state. Data was mapped using IGV.
